# *catena*-Poly[[(1,10-phenanthroline-κ^2^ *N*,*N*′)zinc]-μ-furan-2,5-dicarboxyl­ato-κ^4^ *O* ^2^,*O* ^2′^:*O* ^5^,*O* ^5′^]

**DOI:** 10.1107/S1600536812019836

**Published:** 2012-05-12

**Authors:** Ya-Feng Li, Yue Xu, Xiao-Lin Qin, Wen-Yuan Gao, Yue Gao

**Affiliations:** a School of Chemical Engineering, Changchun University of Technology, Changchun 130012, People’s Republic of China

## Abstract

In the title coordination polymer, [Zn(C_6_H_2_O_5_)(C_12_H_8_N_2_)]_*n*_, an infinite chain is formed along [010] by linking the chelated {Zn(phen)} entities (phen is 1,10-phenanthroline) with two carboxyl­ate groups of the furan-2,5-dicarboxyl­ate ligand. The Zn^II^ atom shows trigonal–prismatic coordination.

## Related literature

For related structures, see: Li, Gao *et al.* (2012 ▶); Li, Xu *et al.* (2012 ▶).
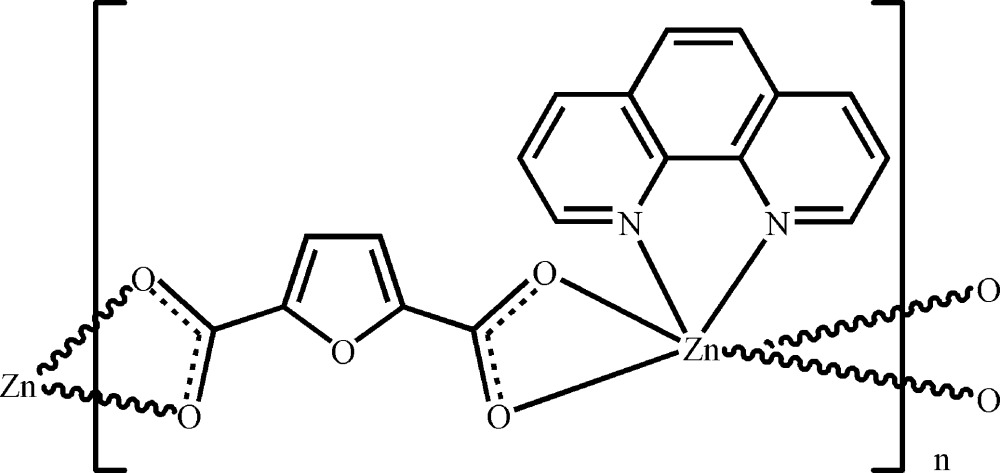


## Experimental

### 

#### Crystal data

[Zn(C_6_H_2_O_5_)(C_12_H_8_N_2_)]
*M* *_r_* = 399.67
Monoclinic, 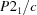

*a* = 5.8725 (10) Å
*b* = 15.013 (3) Å
*c* = 19.241 (8) Å
β = 104.42 (3)°
*V* = 1642.9 (8) Å^3^
*Z* = 4
Mo *K*α radiation
μ = 1.53 mm^−1^
*T* = 293 K
0.16 × 0.13 × 0.12 mm

#### Data collection

Rigaku R-AXIS RAPID diffractometer
Absorption correction: multi-scan (*ABSCOR*; Higashi, 1995 ▶) *T*_min_ = 0.792, *T*_max_ = 0.838
14467 measured reflections
3688 independent reflections
2166 reflections with *I* > 2σ(*I*)
*R*_int_ = 0.066

#### Refinement

*R*[*F* ^2^ > 2σ(*F* ^2^)] = 0.055
*wR*(*F* ^2^) = 0.121
*S* = 1.04
3688 reflections
235 parameters
156 restraints
H-atom parameters constrained
Δρ_max_ = 0.37 e Å^−3^
Δρ_min_ = −0.37 e Å^−3^

### 

Data collection: *PROCESS-AUTO* (Rigaku, 1998 ▶); cell refinement: *PROCESS-AUTO*; data reduction: *CrystalStructure* (Rigaku/MSC, 2002 ▶); program(s) used to solve structure: *SHELXS97* (Sheldrick, 2008 ▶); program(s) used to refine structure: *SHELXL97* (Sheldrick, 2008 ▶); molecular graphics: *DIAMOND* (Brandenburg, 2000 ▶); software used to prepare material for publication: *SHELXL97*.

## Supplementary Material

Crystal structure: contains datablock(s) I, global. DOI: 10.1107/S1600536812019836/ng5269sup1.cif

Structure factors: contains datablock(s) I. DOI: 10.1107/S1600536812019836/ng5269Isup2.hkl

Additional supplementary materials:  crystallographic information; 3D view; checkCIF report

## Figures and Tables

**Fig. 1.**
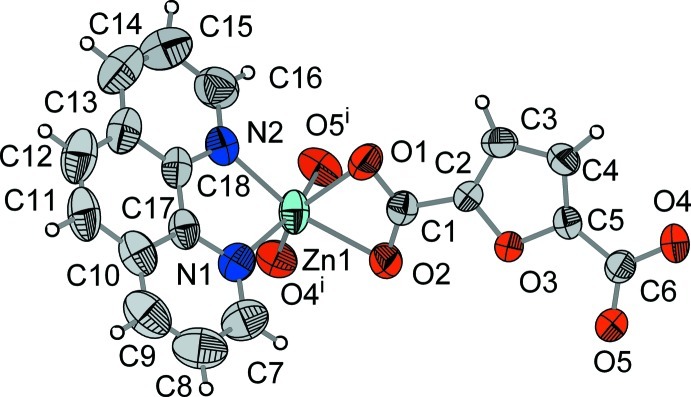
The unit cell of (I), showing the atomic labelling scheme and displacement ellipsoids at the 50% probability level. [Symmetry codes: (i) 1 - x, -0.5 + y, 0.5 - z.]

**Fig. 2.**
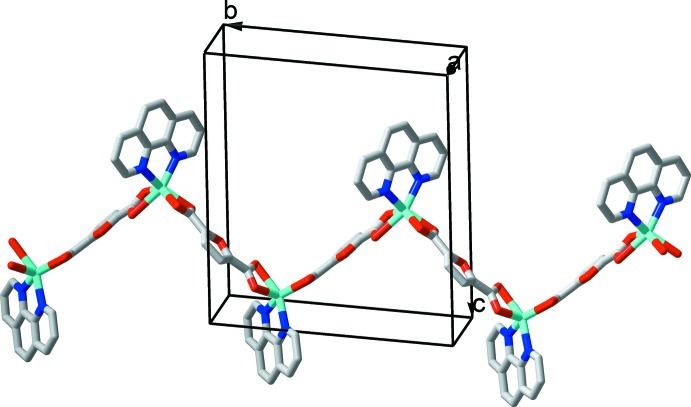
The stick plot of (I), displaying the infinite chain along [010] direction formed by linking the Zn with two carboxyls of furan-2,5-dicarboxylate.

**Fig. 3.**
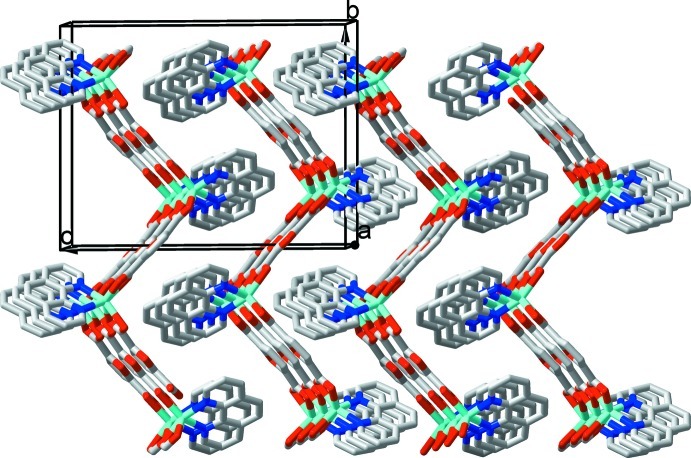
The ball-stick packing diagram of (I). The adjacent chains are piled up along [100] through π-π interactions between C12H8N2 molecules of different chains.

## supplementary crystallographic information

### Comment

Recently, we utilized furan-2,5-dicarboxyl icacid as the ligand to constructed the MOFs (Li, Gao *et al.* 2012; Li, Xu *et al.*, 2012). In this work, a chainlike compound, [Zn(C_12_H_8_N_2_)(C_6_H_2_O_5_)]_n_ (I), is reported.

The asymmetric unit consists of one Zn cation, one furan-2,5-dicarboxylate anion and one C_12_H_8_N_2_ (Fig.1). The Zn cation is coordinated by four carboxylate O atoms, two nitrogen of one C_12_H_8_N_2_, exhibiting triganol prismatic coordination. The furan-2,5-dicarboxylate shows a µ_2_:η^1^,η^1^;η^1^,η^1^ coordinating mode. The dihedral angles of two furan rings *versus*. C_6_H_2_O_5_ ring and two furan rings, which are coordinated to the same Zn cation, are 52.06 (12)°, 62.18 (11)° and 88.27 (12)°, respectively.

TheZn cations are linked by two carboxylate of furan-2,5-dicarboxylate to give rise to an infinite chain (Fig. 2). The adjacent chains are piled up along [100] through π-π interactions between C_12_H_8_N_2_ molecules of different chains. vVan der Waals forces exist between the layers (Fig. 3).

### Experimental

Furan-2,5-dicarboxylic acid (0.0156 g, 0.10 mmol), Zn(NO_3_)_2_^.^6H_2_O (0.0298 g, 0.10 mmol), and C_12_H_8_N_2_ (0.0198, 0.11 mmol) were dissolved in DMF (5 ml, 48 mmol) under stirring. The mixture with molar ratio of 1 (furan-2,5-dicarboxyl acid): 1 (Zn(NO_3_)_2_^.^6H_2_O): 1.1 (C_12_H_8_N_2_): 480 DMI was heated under 120°C for 2 days. Colorless blocks were collected as a single phase.

### Refinement

The carbon H-atoms were placed in calculated positions (C—H (furan ring and phen ring) = 0.93 Å) and were included in the refinement in the riding-model approximation, with *U*_iso_(H) = 1.2Ueq(C).

### Crystal data

**Table:**

[Zn(C_6_H_2_O_5_)(C_12_H_8_N_2_)]	*F*(000) = 808
*M**_r_* = 399.67	*D*_x_ = 1.616 Mg m^−^^3^
Monoclinic, *P*2_1_/*c*	Mo *K*α radiation, λ = 0.71073 Å
Hall symbol: -P 2ybc	Cell parameters from 2000 reflections
*a* = 5.8725 (10) Å	θ = 3.5–27.5°
*b* = 15.013 (3) Å	µ = 1.53 mm^−^^1^
*c* = 19.241 (8) Å	*T* = 293 K
β = 104.42 (3)°	Block, colorless
*V* = 1642.9 (8) Å^3^	0.16 × 0.13 × 0.12 mm
*Z* = 4	

### Data collection

**Table:**

Rigaku R-AXIS RAPID diffractometer	3688 independent reflections
Radiation source: fine-focus sealed tube	2166 reflections with *I* > 2σ(*I*)
Graphite monochromator	*R*_int_ = 0.066
Detector resolution: 10.00 pixels mm^-1^	θ_max_ = 27.5°, θ_min_ = 3.5°
ω scans	*h* = −7→7
Absorption correction: multi-scan (*ABSCOR*; Higashi, 1995)	*k* = −19→19
*T*_min_ = 0.792, *T*_max_ = 0.838	*l* = −24→24
14467 measured reflections	

### Refinement

**Table:**

Refinement on *F*^2^	Primary atom site location: structure-invariant direct methods
Least-squares matrix: full	Secondary atom site location: difference Fourier map
*R*[*F*^2^ > 2σ(*F*^2^)] = 0.055	Hydrogen site location: inferred from neighbouring sites
*wR*(*F*^2^) = 0.121	H-atom parameters constrained
*S* = 1.04	*w* = 1/[σ^2^(*F*_o_^2^) + (0.0305*P*)^2^ + 1.7497*P*] where *P* = (*F*_o_^2^ + 2*F*_c_^2^)/3
3688 reflections	(Δ/σ)_max_ < 0.001
235 parameters	Δρ_max_ = 0.37 e Å^−^^3^
156 restraints	Δρ_min_ = −0.37 e Å^−^^3^

### Special details

**Table:**

Geometry. All e.s.d.'s (except the e.s.d. in the dihedral angle between two l.s. planes) are estimated using the full covariance matrix. The cell e.s.d.'s are taken into account individually in the estimation of e.s.d.'s in distances, angles and torsion angles; correlations between e.s.d.'s in cell parameters are only used when they are defined by crystal symmetry. An approximate (isotropic) treatment of cell e.s.d.'s is used for estimating e.s.d.'s involving l.s. planes.
Refinement. Refinement of *F*^2^ against ALL reflections. The weighted *R*-factor *wR* and goodness of fit *S* are based on *F*^2^, conventional *R*-factors *R* are based on *F*, with *F* set to zero for negative *F*^2^. The threshold expression of *F*^2^ > σ(*F*^2^) is used only for calculating *R*-factors(gt) *etc*. and is not relevant to the choice of reflections for refinement. *R*-factors based on *F*^2^ are statistically about twice as large as those based on *F*, and *R*- factors based on ALL data will be even larger.

### Fractional atomic coordinates and isotropic or equivalent isotropic displacement parameters (Å^2^)

**Table:**

	*x*	*y*	*z*	*U*_iso_*/*U*_eq_	
Zn1	0.35240 (10)	0.25875 (4)	0.08732 (3)	0.0611 (2)	
O1	0.6391 (5)	0.3452 (2)	0.11677 (17)	0.0704 (9)	
O2	0.3330 (5)	0.3794 (2)	0.15596 (16)	0.0662 (9)	
O3	0.5760 (4)	0.51458 (17)	0.23919 (13)	0.0448 (6)	
O4	0.8463 (5)	0.6748 (2)	0.36985 (15)	0.0636 (8)	
O5	0.4722 (5)	0.6501 (2)	0.32328 (16)	0.0686 (9)	
N1	0.1045 (7)	0.3030 (3)	−0.00407 (19)	0.0614 (9)	
N2	0.4670 (6)	0.1904 (3)	0.00778 (18)	0.0606 (10)	
C1	0.5395 (7)	0.3928 (3)	0.1538 (2)	0.0503 (10)	
C2	0.6835 (6)	0.4618 (3)	0.1988 (2)	0.0437 (9)	
C3	0.9155 (7)	0.4798 (3)	0.2139 (2)	0.0565 (11)	
H3	1.0259	0.4532	0.1933	0.068*	
C4	0.9582 (7)	0.5469 (3)	0.2670 (2)	0.0544 (11)	
H4	1.1028	0.5724	0.2886	0.065*	
C5	0.7492 (6)	0.5669 (3)	0.28057 (19)	0.0444 (9)	
C6	0.6828 (7)	0.6332 (3)	0.3271 (2)	0.0484 (10)	
C7	−0.0749 (10)	0.3570 (4)	−0.0079 (3)	0.0833 (16)	
H7	−0.0992	0.3816	0.0340	0.100*	
C8	−0.2319 (10)	0.3783 (4)	−0.0746 (4)	0.0997 (19)	
H8	−0.3571	0.4170	−0.0767	0.120*	
C9	−0.1958 (10)	0.3409 (4)	−0.1357 (3)	0.0943 (19)	
H9	−0.2991	0.3539	−0.1797	0.113*	
C10	−0.0086 (9)	0.2843 (4)	−0.1334 (3)	0.0711 (14)	
C11	0.0446 (11)	0.2424 (4)	−0.1952 (2)	0.0849 (17)	
H11	−0.0532	0.2524	−0.2405	0.102*	
C12	0.2292 (12)	0.1902 (4)	−0.1882 (3)	0.0942 (18)	
H12	0.2631	0.1667	−0.2293	0.113*	
C13	0.3771 (10)	0.1689 (4)	−0.1206 (3)	0.0757 (14)	
C14	0.5738 (13)	0.1132 (5)	−0.1103 (4)	0.107 (2)	
H14	0.6125	0.0874	−0.1497	0.129*	
C15	0.7072 (12)	0.0968 (5)	−0.0442 (4)	0.112 (2)	
H15	0.8534	0.0666	−0.0303	0.135*	
C16	0.6508 (9)	0.1371 (4)	0.0144 (3)	0.0880 (17)	
H16	0.7459	0.1262	0.0601	0.106*	
C17	0.1378 (7)	0.2662 (3)	−0.0654 (2)	0.0542 (11)	
C18	0.3306 (8)	0.2074 (3)	−0.0590 (2)	0.0574 (11)	

### Atomic displacement parameters (Å^2^)

**Table:**

	*U*^11^	*U*^22^	*U*^33^	*U*^12^	*U*^13^	*U*^23^
Zn1	0.0831 (4)	0.0578 (3)	0.0401 (3)	−0.0158 (3)	0.0106 (2)	−0.0017 (2)
O1	0.0689 (19)	0.066 (2)	0.082 (2)	−0.0051 (16)	0.0292 (17)	−0.0279 (17)
O2	0.0538 (18)	0.079 (2)	0.068 (2)	−0.0167 (15)	0.0205 (15)	−0.0220 (16)
O3	0.0420 (14)	0.0468 (16)	0.0452 (14)	−0.0007 (11)	0.0099 (11)	−0.0086 (12)
O4	0.0610 (18)	0.064 (2)	0.0605 (18)	−0.0104 (15)	0.0055 (14)	−0.0212 (15)
O5	0.0543 (18)	0.082 (2)	0.0668 (19)	0.0076 (16)	0.0109 (15)	−0.0272 (17)
N1	0.071 (2)	0.061 (2)	0.054 (2)	0.0000 (19)	0.0195 (18)	0.0055 (18)
N2	0.062 (2)	0.063 (2)	0.053 (2)	−0.0052 (19)	0.0054 (18)	−0.0020 (18)
C1	0.056 (2)	0.048 (2)	0.045 (2)	0.0009 (19)	0.0110 (19)	−0.0012 (18)
C2	0.046 (2)	0.043 (2)	0.044 (2)	0.0021 (16)	0.0130 (17)	−0.0064 (17)
C3	0.045 (2)	0.058 (3)	0.068 (3)	0.0036 (19)	0.018 (2)	−0.004 (2)
C4	0.042 (2)	0.055 (3)	0.063 (3)	−0.0041 (18)	0.008 (2)	−0.007 (2)
C5	0.045 (2)	0.042 (2)	0.043 (2)	−0.0048 (17)	0.0061 (17)	−0.0030 (16)
C6	0.052 (2)	0.047 (2)	0.045 (2)	0.0031 (18)	0.0118 (19)	−0.0031 (17)
C7	0.092 (4)	0.085 (4)	0.083 (4)	0.008 (3)	0.040 (3)	0.012 (3)
C8	0.083 (4)	0.101 (5)	0.117 (5)	0.020 (3)	0.028 (4)	0.033 (4)
C9	0.082 (4)	0.109 (5)	0.080 (4)	−0.005 (3)	−0.002 (3)	0.031 (3)
C10	0.075 (3)	0.073 (3)	0.057 (3)	−0.015 (3)	0.002 (2)	0.016 (2)
C11	0.113 (4)	0.096 (4)	0.037 (2)	−0.030 (3)	0.002 (3)	0.001 (3)
C12	0.141 (5)	0.094 (4)	0.051 (3)	−0.019 (4)	0.030 (3)	−0.010 (3)
C13	0.101 (4)	0.064 (3)	0.065 (3)	−0.014 (3)	0.027 (3)	−0.012 (2)
C14	0.137 (5)	0.091 (4)	0.108 (5)	0.007 (4)	0.056 (4)	−0.022 (4)
C15	0.110 (5)	0.107 (5)	0.129 (5)	0.028 (4)	0.046 (4)	−0.001 (4)
C16	0.076 (3)	0.086 (4)	0.096 (4)	0.009 (3)	0.009 (3)	0.003 (3)
C17	0.065 (3)	0.057 (3)	0.039 (2)	−0.014 (2)	0.0092 (19)	0.0027 (18)
C18	0.072 (3)	0.056 (3)	0.045 (2)	−0.016 (2)	0.015 (2)	−0.0059 (19)

### Geometric parameters (Å, º)

**Table:**

Zn1—O4^i^	2.027 (3)	C5—C6	1.455 (5)
Zn1—N2	2.089 (4)	C7—C8	1.418 (7)
Zn1—O1	2.090 (3)	C7—H7	0.9300
Zn1—N1	2.092 (4)	C8—C9	1.367 (8)
Zn1—O2	2.261 (3)	C8—H8	0.9300
Zn1—O5^i^	2.408 (3)	C9—C10	1.381 (8)
O1—C1	1.252 (5)	C9—H9	0.9300
O2—C1	1.240 (5)	C10—C17	1.401 (6)
O3—C2	1.369 (4)	C10—C11	1.447 (7)
O3—C5	1.372 (4)	C11—C12	1.317 (8)
O4—C6	1.263 (4)	C11—H11	0.9300
O5—C6	1.246 (4)	C12—C13	1.410 (7)
N1—C7	1.316 (6)	C12—H12	0.9300
N1—C17	1.361 (5)	C13—C14	1.399 (8)
N2—C16	1.324 (6)	C13—C18	1.405 (6)
N2—C18	1.358 (5)	C14—C15	1.341 (9)
C1—C2	1.475 (5)	C14—H14	0.9300
C2—C3	1.347 (5)	C15—C16	1.390 (8)
C3—C4	1.413 (6)	C15—H15	0.9487
C3—H3	0.9300	C16—H16	0.9300
C4—C5	1.351 (5)	C17—C18	1.416 (6)
C4—H4	0.9300		
			
O4^i^—Zn1—N2	108.37 (14)	C4—C3—H3	126.7
O4^i^—Zn1—O1	141.51 (13)	C5—C4—C3	107.0 (4)
N2—Zn1—O1	96.85 (13)	C5—C4—H4	126.5
O4^i^—Zn1—N1	100.88 (13)	C3—C4—H4	126.5
N2—Zn1—N1	79.86 (15)	C4—C5—O3	109.8 (3)
O1—Zn1—N1	112.06 (14)	C4—C5—C6	131.8 (4)
O4^i^—Zn1—O2	98.21 (12)	O3—C5—C6	118.4 (3)
N2—Zn1—O2	153.40 (13)	O5—C6—O4	121.3 (4)
O1—Zn1—O2	59.89 (11)	O5—C6—C5	121.1 (4)
N1—Zn1—O2	96.36 (14)	O4—C6—C5	117.5 (3)
O4^i^—Zn1—O5^i^	58.36 (10)	O5—C6—Zn1^ii^	69.4 (2)
N2—Zn1—O5^i^	91.83 (13)	O4—C6—Zn1^ii^	51.9 (2)
O1—Zn1—O5^i^	93.13 (12)	C5—C6—Zn1^ii^	169.1 (3)
N1—Zn1—O5^i^	154.14 (13)	N1—C7—C8	121.4 (5)
O2—Zn1—O5^i^	101.54 (12)	N1—C7—H7	119.3
O4^i^—Zn1—C1	121.27 (13)	C8—C7—H7	119.3
N2—Zn1—C1	126.34 (14)	C9—C8—C7	118.8 (6)
O1—Zn1—C1	30.16 (12)	C9—C8—H8	120.6
N1—Zn1—C1	107.51 (14)	C7—C8—H8	120.6
O2—Zn1—C1	29.78 (11)	C8—C9—C10	121.1 (5)
O5^i^—Zn1—C1	97.18 (12)	C8—C9—H9	119.4
O4^i^—Zn1—C6^i^	29.39 (11)	C10—C9—H9	119.4
N2—Zn1—C6^i^	101.69 (14)	C9—C10—C17	116.6 (5)
O1—Zn1—C6^i^	118.33 (13)	C9—C10—C11	125.1 (5)
N1—Zn1—C6^i^	128.86 (14)	C17—C10—C11	118.3 (5)
O2—Zn1—C6^i^	101.04 (13)	C12—C11—C10	121.1 (5)
O5^i^—Zn1—C6^i^	28.98 (10)	C12—C11—H11	119.4
C1—Zn1—C6^i^	111.29 (13)	C10—C11—H11	119.4
C1—O1—Zn1	92.8 (2)	C11—C12—C13	122.1 (6)
C1—O2—Zn1	85.3 (2)	C11—C12—H12	119.0
C2—O3—C5	106.3 (3)	C13—C12—H12	119.0
C6—O4—Zn1^ii^	98.7 (2)	C14—C13—C18	116.9 (5)
C6—O5—Zn1^ii^	81.6 (2)	C14—C13—C12	124.3 (6)
C7—N1—C17	119.2 (4)	C18—C13—C12	118.7 (5)
C7—N1—Zn1	128.5 (4)	C15—C14—C13	120.8 (6)
C17—N1—Zn1	112.3 (3)	C15—C14—H14	119.6
C16—N2—C18	118.7 (4)	C13—C14—H14	119.6
C16—N2—Zn1	129.0 (4)	C14—C15—C16	119.2 (6)
C18—N2—Zn1	112.3 (3)	C14—C15—H15	129.0
O2—C1—O1	121.8 (4)	C16—C15—H15	111.2
O2—C1—C2	121.1 (4)	N2—C16—C15	122.5 (6)
O1—C1—C2	117.0 (4)	N2—C16—H16	118.7
O2—C1—Zn1	64.9 (2)	C15—C16—H16	118.7
O1—C1—Zn1	57.0 (2)	N1—C17—C10	122.9 (4)
C2—C1—Zn1	170.2 (3)	N1—C17—C18	117.5 (4)
C3—C2—O3	110.2 (3)	C10—C17—C18	119.6 (4)
C3—C2—C1	132.0 (4)	N2—C18—C13	121.8 (5)
O3—C2—C1	117.5 (3)	N2—C18—C17	118.0 (4)
C2—C3—C4	106.7 (4)	C13—C18—C17	120.1 (4)
C2—C3—H3	126.7		
			
O4^i^—Zn1—O1—C1	−59.7 (3)	O2—C1—C2—C3	168.7 (4)
N2—Zn1—O1—C1	168.9 (3)	O1—C1—C2—C3	−7.3 (7)
N1—Zn1—O1—C1	87.1 (3)	O2—C1—C2—O3	−4.5 (6)
O2—Zn1—O1—C1	2.6 (2)	O1—C1—C2—O3	179.6 (4)
O5^i^—Zn1—O1—C1	−98.9 (3)	O3—C2—C3—C4	0.5 (5)
C6^i^—Zn1—O1—C1	−83.9 (3)	C1—C2—C3—C4	−173.0 (4)
O4^i^—Zn1—O2—C1	143.5 (2)	C2—C3—C4—C5	−0.9 (5)
N2—Zn1—O2—C1	−34.5 (4)	C3—C4—C5—O3	0.9 (5)
O1—Zn1—O2—C1	−2.6 (2)	C3—C4—C5—C6	−176.6 (4)
N1—Zn1—O2—C1	−114.5 (3)	C2—O3—C5—C4	−0.6 (4)
O5^i^—Zn1—O2—C1	84.3 (3)	C2—O3—C5—C6	177.3 (3)
C6^i^—Zn1—O2—C1	113.8 (3)	Zn1^ii^—O5—C6—O4	1.0 (4)
O4^i^—Zn1—N1—C7	71.3 (4)	Zn1^ii^—O5—C6—C5	−176.7 (4)
N2—Zn1—N1—C7	178.3 (4)	Zn1^ii^—O4—C6—O5	−1.2 (5)
O1—Zn1—N1—C7	−88.4 (4)	Zn1^ii^—O4—C6—C5	176.6 (3)
O2—Zn1—N1—C7	−28.3 (4)	C4—C5—C6—O5	168.5 (4)
O5^i^—Zn1—N1—C7	105.4 (5)	O3—C5—C6—O5	−8.9 (6)
C1—Zn1—N1—C7	−56.6 (4)	C4—C5—C6—O4	−9.3 (7)
C6^i^—Zn1—N1—C7	81.4 (5)	O3—C5—C6—O4	173.3 (3)
O4^i^—Zn1—N1—C17	−106.0 (3)	C4—C5—C6—Zn1^ii^	4.8 (19)
N2—Zn1—N1—C17	1.0 (3)	O3—C5—C6—Zn1^ii^	−172.5 (13)
O1—Zn1—N1—C17	94.3 (3)	C17—N1—C7—C8	−0.8 (7)
O2—Zn1—N1—C17	154.4 (3)	Zn1—N1—C7—C8	−178.0 (4)
O5^i^—Zn1—N1—C17	−71.9 (4)	N1—C7—C8—C9	0.7 (9)
C1—Zn1—N1—C17	126.1 (3)	C7—C8—C9—C10	−0.9 (9)
C6^i^—Zn1—N1—C17	−95.9 (3)	C8—C9—C10—C17	1.2 (8)
O4^i^—Zn1—N2—C16	−84.1 (4)	C8—C9—C10—C11	−179.5 (5)
O1—Zn1—N2—C16	66.4 (4)	C9—C10—C11—C12	179.0 (6)
N1—Zn1—N2—C16	177.7 (4)	C17—C10—C11—C12	−1.7 (8)
O2—Zn1—N2—C16	93.7 (5)	C10—C11—C12—C13	3.3 (9)
O5^i^—Zn1—N2—C16	−27.0 (4)	C11—C12—C13—C14	179.4 (6)
C1—Zn1—N2—C16	73.3 (5)	C11—C12—C13—C18	−2.5 (9)
C6^i^—Zn1—N2—C16	−54.5 (4)	C18—C13—C14—C15	1.2 (9)
O4^i^—Zn1—N2—C18	97.8 (3)	C12—C13—C14—C15	179.4 (6)
O1—Zn1—N2—C18	−111.7 (3)	C13—C14—C15—C16	−1.2 (11)
N1—Zn1—N2—C18	−0.4 (3)	C18—N2—C16—C15	−1.1 (8)
O2—Zn1—N2—C18	−84.3 (4)	Zn1—N2—C16—C15	−179.1 (5)
O5^i^—Zn1—N2—C18	154.9 (3)	C14—C15—C16—N2	1.2 (10)
C1—Zn1—N2—C18	−104.8 (3)	C7—N1—C17—C10	1.2 (7)
C6^i^—Zn1—N2—C18	127.5 (3)	Zn1—N1—C17—C10	178.8 (3)
Zn1—O2—C1—O1	4.5 (4)	C7—N1—C17—C18	−179.0 (4)
Zn1—O2—C1—C2	−171.2 (4)	Zn1—N1—C17—C18	−1.4 (5)
Zn1—O1—C1—O2	−4.9 (4)	C9—C10—C17—N1	−1.4 (7)
Zn1—O1—C1—C2	171.0 (3)	C11—C10—C17—N1	179.2 (4)
O4^i^—Zn1—C1—O2	−43.5 (3)	C9—C10—C17—C18	178.9 (4)
N2—Zn1—C1—O2	161.7 (2)	C11—C10—C17—C18	−0.5 (6)
O1—Zn1—C1—O2	175.4 (4)	C16—N2—C18—C13	1.2 (7)
N1—Zn1—C1—O2	71.5 (3)	Zn1—N2—C18—C13	179.5 (3)
O5^i^—Zn1—C1—O2	−100.7 (2)	C16—N2—C18—C17	−178.5 (4)
C6^i^—Zn1—C1—O2	−74.5 (3)	Zn1—N2—C18—C17	−0.2 (5)
O4^i^—Zn1—C1—O1	141.0 (2)	C14—C13—C18—N2	−1.2 (7)
N2—Zn1—C1—O1	−13.8 (3)	C12—C13—C18—N2	−179.5 (5)
N1—Zn1—C1—O1	−103.9 (3)	C14—C13—C18—C17	178.5 (5)
O2—Zn1—C1—O1	−175.4 (4)	C12—C13—C18—C17	0.2 (7)
O5^i^—Zn1—C1—O1	83.8 (3)	N1—C17—C18—N2	1.1 (6)
C6^i^—Zn1—C1—O1	110.1 (3)	C10—C17—C18—N2	−179.1 (4)
C5—O3—C2—C3	0.0 (4)	N1—C17—C18—C13	−178.6 (4)
C5—O3—C2—C1	174.6 (3)	C10—C17—C18—C13	1.2 (6)

Symmetry codes: (i) −*x*+1, *y*−1/2, −*z*+1/2; (ii) −*x*+1, *y*+1/2, −*z*+1/2.
